# The brain-specific splice variant of the CDC42 GTPase works together with the kinase ACK to downregulate the EGF receptor in promoting neurogenesis

**DOI:** 10.1016/j.jbc.2022.102564

**Published:** 2022-10-05

**Authors:** Makoto Endo, Richard A. Cerione

**Affiliations:** 1 Department of Molecular Medicine, Cornell University, Ithaca, New York, USA; 2 Department of Chemistry and Chemical Biology, Cornell University, Ithaca, New York, USA

**Keywords:** CDC42, ACK, EGFR, mTOR, neurogenesis, autophagy, 3MA, 3-methyladenine, CQ, chloroquine, CNS, central nervous system, EGFR, EGF receptor, FBS, fetal bovine serum, HVR, hypervariable region, PIP_2_, phosphatidylinositol (4,5)-bisphosphate, PIP_3_, phosphatidylinositol (3,4,5)-triphosphate, PM, plasma membrane, RA, retinoic acid, RIPA, radioimmunoprecipitation assay

## Abstract

The small GTPase CDC42 plays essential roles in neurogenesis and brain development. Previously, we showed that a CDC42 splice variant that has a ubiquitous tissue distribution specifically stimulates the formation of neural progenitor cells, whereas a brain-specific CDC42 variant, CDC42b, is essential for promoting the transition of neural progenitor cells to neurons. These specific roles of CDC42 and CDC42b in neurogenesis are ascribed to their opposing effects on mTORC1 activity. Specifically, the ubiquitous form of CDC42 stimulates mTORC1 activity and thereby upregulates tissue-specific transcription factors that are essential for neuroprogenitor formation, whereas CDC42b works together with activated CDC42-associated kinase (ACK) to downregulate mTOR expression. Here, we demonstrate that the EGF receptor (EGFR) is an additional and important target of CDC42b and ACK, which is downregulated by their combined actions in promoting neurogenesis. The activation status of the EGFR determines the timing by which neural progenitor cells derived from P19 embryonal carcinoma terminally differentiate into neurons. By promoting EGFR degradation, we found that CDC42b and ACK stimulate autophagy, which protects emerging neurons from apoptosis and helps trigger neural progenitor cells to differentiate into neurons. Moreover, our results reveal that CDC42b is localized in phosphatidylinositol (3,4,5)-triphosphate–enriched microdomains on the plasma membrane, mediated through its polybasic sequence ^185^KRK^187^, which is essential for determining its distinct functions. Overall, these findings now highlight a molecular mechanism by which CDC42b and ACK regulate neuronal differentiation and provide new insights into the functional interplay between EGFR degradation and autophagy that occurs during embryonic neurogenesis.

The Rho family small GTPase CDC42 functions as a molecular switch controlling numerous cellular activities, including cytokinesis, cell cycle progression, polarity, morphology, motility, and transformation (1, 2, 3, 4). The cellular activities of CDC42 are indispensable for embryogenesis, as represented by the early embryonic lethality in mice as a result of knocking-out CDC42 (5). Tissue-specific conditional KO mice also highlight the importance of CDC42 in embryonic organogenesis and tissue homeostasis (6, 7). A particularly striking example is that depletion of CDC42 causes serious defects in central nervous system (CNS) development (8, 9, 10). When CDC42 is knocked out in apical stem/progenitor cells in mouse telencephalons, these cells exhibit an inability to maintain cell polarity and epithelial structures and ultimately fail to adopt their proper cell fate (8, 9, 10). The brain-specific KO of CDC42 eventually causes hydrocephaly or holoprosencephaly (8, 10), demonstrating the importance of CDC42 in establishing and maintaining intricate tissue structures during CNS development. Considering the wide range of regulatory functions provided by CDC42, it is reasonable to assume that CDC42 triggers multiple signals to influence the many facets of neural stem/progenitor cell development during embryogenesis. Indeed, we previously showed that CDC42 is involved in the cell fate determination of Nestin-positive neural progenitor cells by controlling the expression of tissue-specific transcription factors, using embryonal carcinoma P19 cells as a model system (11). Upon stimulation with retinoic acid (RA), FGF-dependent and Delta/Notch-dependent signaling pathways are upregulated and activate CDC42 during the cell lineage specification phase of P19 cells. Activated CDC42 controls mTOR activation status, leading to the upregulation of HES5 and PAX6, which are the key transcription factors for determining the embryonic apical neural stem/progenitor cell fate. When CDC42 is overexpressed or hyperactivated by exogenous expression of WT CDC42 or a constitutively active CDC42 mutant, P19 cells spontaneously differentiate into Nestin-positive neural progenitor cells even in the absence of induction but also lose their ability to terminally differentiate into postmitotic neurons. Although the ectopic expression of CDC42 inhibits terminal differentiation, the expression and activation levels of endogenous CDC42 continue to increase during the time window of terminal differentiation into neurons, even after the neural cell lineage specification phase (11). These results lead to a fundamental question: why does ectopically expressed CDC42 halt differentiation at the neural progenitor stage in P19 cells, while the upregulation of endogenous CDC42 fails to inhibit terminal differentiation into neurons.

In the following study, we have addressed this paradoxical question by showing how two splice variants of CDC42 work to coordinate neurogenesis in embryonic cells (12). CDC42 is conserved throughout eukaryotic evolution from yeast to humans. Although eukaryotes before chordates express only one form of CDC42, chordates including vertebrates express an additional CDC42 splice variant generated by alternative splicing (13, 14). In mammals, this CDC42 splice variant is expressed specifically in the brain (therefore, hereafter designated CDC42b), whereas the canonical CDC42 is ubiquitously expressed in all organs (15, 16, 17, 18). Although numerous studies have been conducted on the canonical form of CDC42, much less is known about CDC42b. These two forms of CDC42 share a common GTPase domain, as well as the main regulator/effector–binding sites, with their only differences being within the carboxyl terminal nine amino acid residues. Small GTPases are single globular domain proteins with distinct stretches of carboxyl terminal residues (called hypervariable regions, HVRs). Despite the HVRs of small GTPases being essential for their subcellular localization (19, 20, 21), it was commonly assumed that CDC42 and CDC42b were functionally redundant, given that their GTPase domains are identical and that both human CDC42 splice variants can suppress *cdc42-1* mutation in budding yeast (15, 22). However, previously we found that the slight differences in their HVRs were sufficient to enable the two CDC42 splice variants to play distinct roles in mouse embryonic neurogenesis (12). Specifically, while both CDC42 proteins are equally capable of binding to a downstream signaling target, Activated CDC42-associated Kinase (ACK), only CDC42b works together with ACK to promote the ubiquitination and degradation of mTOR, which leads to the downregulation of PAX6 and the promotion of neurogenesis in P19 embryonal carcinoma, as well as in E14 mouse embryonic stem cells (12).

ACK functions as a dual kinase, phosphorylating both tyrosine and serine/threonine residues (23, 24, 25), and is highly expressed in the CNS (26). It has been suggested to interact with the EGF receptor (EGFR) and to be involved in promoting EGFR ubiquitination and degradation (27, 28, 29, 30). ACK is also involved in intracellular trafficking, delivering EGFRs to autophagosomes, as mediated by interactions with the autophagy adapter proteins p62 and NBR1 (31). However, the biological functions of ACK in autophagosomes and in the noncanonical trafficking of EGFRs remain unanswered. Since both EGFR signaling and autophagy play essential roles in neurogenesis, ACK may be involved in neuronal development by regulating EGFR homeostasis and autophagy.

One of the most important but unanswered questions regarding the CDC42 splicing variants is how they obtain distinct abilities, with the slight differences in their HVRs. The HVRs of small GTPases contain polybasic sequences and CAAX motifs, which are essential for determining their membrane localizations (4, 32, 33, 34). Polybasic sequences interact with phosphoinositides, while CAAX motifs are subject to lipid modifications (4, 32, 33, 34). Since CDC42 splice variants are different in both their polybasic sequences and CAAX motifs, they are assumed to exhibit distinct subcellular localizations, leading to different biological outcomes. Indeed, only CDC42b but not CDC42 has been shown to be subject to palmitoylation in its CAAX motif, thereby distributed to and involved in the formation of dendritic spines in neurons (35, 36, 37). While the functions of the CAAX motif of CDC42b are well established, however, those of the polybasic sequence remain unexplored. Previously, we showed that the diarginine motif (R186 and R187) in the polybasic sequence of CDC42 binds to phosphatidylinositol (4,5)-bisphosphate (PIP_2_), which is essential for the transforming ability of the constitutively active CDC42(F28L) mutant (38). Given the differences of polybasic sequences between CDC42 splice variants, it is possible that CDC42b exhibits different interactions with phosphoinositides from CDC42, thereby obtaining distinct subcellular localizations and biological functions.

Here, we now show that CDC42b and ACK promote the ubiquitination and degradation of EGFRs as well as mTOR. By promoting the degradation of EGFRs, CDC42b and ACK are able to induce autophagy, and thus protect emerging neurons from apoptosis, and promote the transition of proliferative neural progenitor cells into postmitotic neurons. Furthermore, we show that the polybasic sequence ^185^KRK^187^ in the HVR of CDC42b is essential for the interaction with phosphatidylinositol (3,4,5)-triphosphate (PIP_3_), thereby contributing to the distinct subcellular localization and functions of CDC42b. Our results shed light on the actions of CDC42b and ACK in embryonic neurogenesis and provide new insights into the functional connection between EGFR and autophagic activities during neuronal differentiation.

## Results

### CDC42b and ACK promote EGFR ubiquitination and degradation

**Figure 1:**
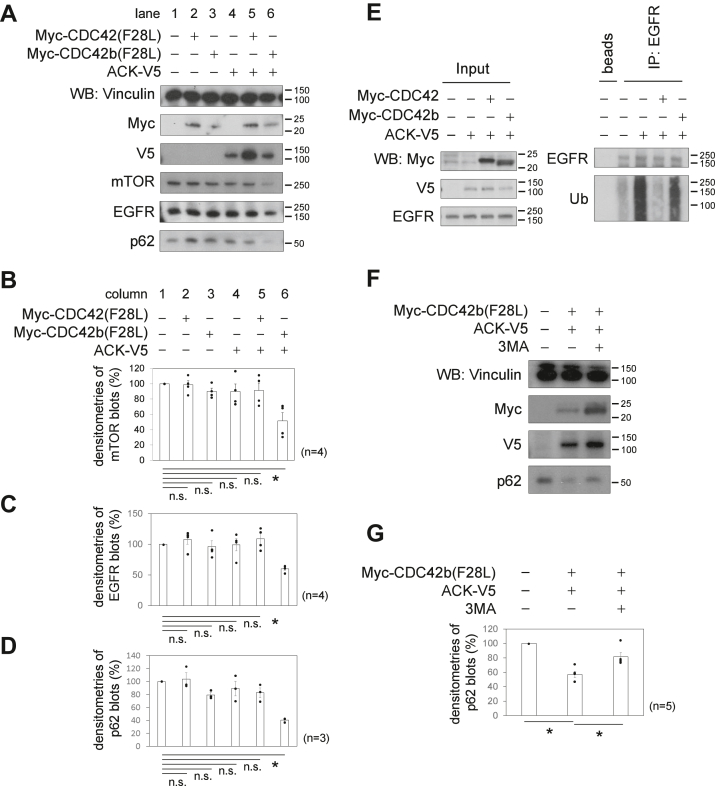
**CDC42b and ACK promote EGFR ubiquitination and degradation.***A*, immunoblotting images showing the expression levels of mTOR, EGFR, and p62 in MDA-MB-231 cells, in the presence or absence of ACK-V5 and/or Myc-tagged constitutively active mutants of the two CDC42 splice variants. Vinculin served as a loading control. *Black bars* and numbers beside the panels indicate the positions and molecular sizes (kDa) of molecular markers. Histograms quantifying the relative amounts of mTOR (*B*), EGFR (*C*), and p62 (*D*), based on densitometry. *E*, immunoblotting images showing the ubiquitination status of the EGFR in HeLa cells in the presence of Myc-tagged CDC42 splice variants and V5-tagged ACK (ACK-V5). EGFRs were immunoprecipitated using an anti-EGFR antibody, and their ubiquitination levels were detected with an antiubiquitin antibody. *F*, immunoblotting images showing the expression levels of p62 in 231 cells expressing ACK-V5 and Myc-CDC42b(F28L), in the presence or absence of 3MA. Histograms quantifying the relative levels of p62 (*G*). Error bars indicate SEM (n = 4 in *B* and *C*, n = 3 in *D*, and n = 5 in *F*). Significance of differences is indicated by n.s. (not significant, *p* > 0.05) and ∗ (*p* < 0.05) using a *t* test (*B*–*D*) or a tukey’s test (*G*). 3MA, 3-methyladenine; EGFR, EGF receptor.

Although ACK has been suggested to promote the ubiquitination and degradation of EGFRs (27, 28, 29, 30), it remains unknown how its binding partners, the two CDC42 splice variants, affect these activities. We then set out to investigate the effects of the CDC42 splice variants and ACK on EGFR homeostasis, using non-neural epithelial cell lines. The individual expression of the constitutively active mutants, Myc-tagged CDC42 (Myc-CDC42(F28L)), Myc-CDC42b(F28L), or the carboxyl terminally V5-tagged ACK (ACK-V5) did not affect the expression of either mTOR or the EGFR in MDA-MB-231 breast cancer cells (Fig. 1*A*, lanes 1–4, Fig. 1, *B* and *C*, columns 1–4). As shown previously, ACK-V5 significantly reduced the expression level of mTOR only when coexpressed with Myc-CDC42b(F28L) but not with Myc-CDC42(F28L) (Fig. 1*A*, lanes 5 and 6, and Fig. 1*B*, columns 5 and 6). Interestingly, the coexpression of Myc-CDC42b(F28L) and ACK-V5 also significantly reduced EGFR expression, whereas Myc-CDC42(F28L) and ACK-V5 did not.

We next examined the ubiquitination status of the EGFR in HeLa cells in the presence or absence of the CDC42 splice variants and ACK. Immunoprecipitated EGFR showed a significantly higher extent of ubiquitination in cells expressing ACK-V5, compared with vector control cells (Fig. 1*E*). The coexpression of Myc-CDC42 with ACK-V5 markedly reduced the level of EGFR ubiquitination, while the coexpression of Myc-CDC42b with ACK-V5 did not change the effects caused by ACK-V5 alone (Fig. 1*E*). These results suggest that overexpression of ACK is sufficient for promoting EGFR ubiquitination and that CDC42, but not CDC42b, suppresses the actions of ACK.

### CDC42b and ACK promote autophagy

Autophagy has been considered as a nonselective process targeting cytosolic proteins and organelles for degradation when nutrients are deprived (39). However, recent studies suggest that some types of autophagic activities selectively degrade protein substrates or damaged organelles, as mediated through the actions of autophagic adapter proteins, including p62 and NBR1 (39). Although ACK has been shown to interact and be colocalized with p62 and NBR1 in autophagosomes (31), it remains unknown whether these interactions influence autophagy. The adapter protein p62 functions as a central hub for selective autophagy by acting to connect ubiquitinated proteins to the phagophore (a precursor of the autophagosome), and p62 itself is subject to autophagic protein degradation (39). MDA-MB-231 cells express a moderate level of p62 in normal culture conditions (Fig. 1*A*, lane 1). The individual expression of either Myc-CDC42(F28L), Myc-CDC42b(F28L), or ACK-V5 did not significantly affect the expression levels of p62 in 231 cells (Fig. 1*A*, lanes 2–4, and Fig. 1*D*, columns 2–4). However, the coexpression of Myc-CDC42b(F28L) and ACK-V5 significantly reduced the expression level of p62, whereas that of Myc-CDC42(F28L) and ACK-V5 did not (Fig. 1*A*, lanes 5 and 6, and Fig. 1*D*, columns 5 and 6). The reduction in p62 expression caused by the coexpression of Myc-CDC42b(F28L) and ACK-V5 was attenuated upon treatment with 3-methyladenine (3MA), which inhibits type III PI-3 kinases and thereby autophagosome formation (Fig. 1, *F* and *G*). Together, these results suggest that CDC42b and ACK promote the degradation of p62 in an autophagy-dependent manner.

To further assess the effects of CDC42b and ACK on autophagic activities, we used an aggregation-prone polyglutamine expansion-tagged GFP construct (HA-Q79-GFP) (40). The accumulation of protein aggregates such as amyloid-beta and α-synuclein has been suggested to underlie the pathogenesis of neurodegenerative diseases. These cytosolic protein aggregates are not processed by the proteasome-dependent protein degradation system but are more efficiently cleared by autophagy. Control GFP did not show specific localization nor did it significantly affect cell survival during the time window that we tested (48 h) (Fig. S1, top panels). In 9 to 12 h after transfection into COS7 cells, HA-Q79-GFP was distributed uniformly throughout the entire cell showing a diffuse fluorescent signal. However, during later time points (15–18 h), it began to exhibit intense punctate fluorescent signals (Fig. S1, bottom panels), suggesting that protein aggregates were formed. In 24 to 48 h, virtually all of the cells expressing HA-Q79-GFP underwent apoptosis (data not shown).

**Figure 2:**
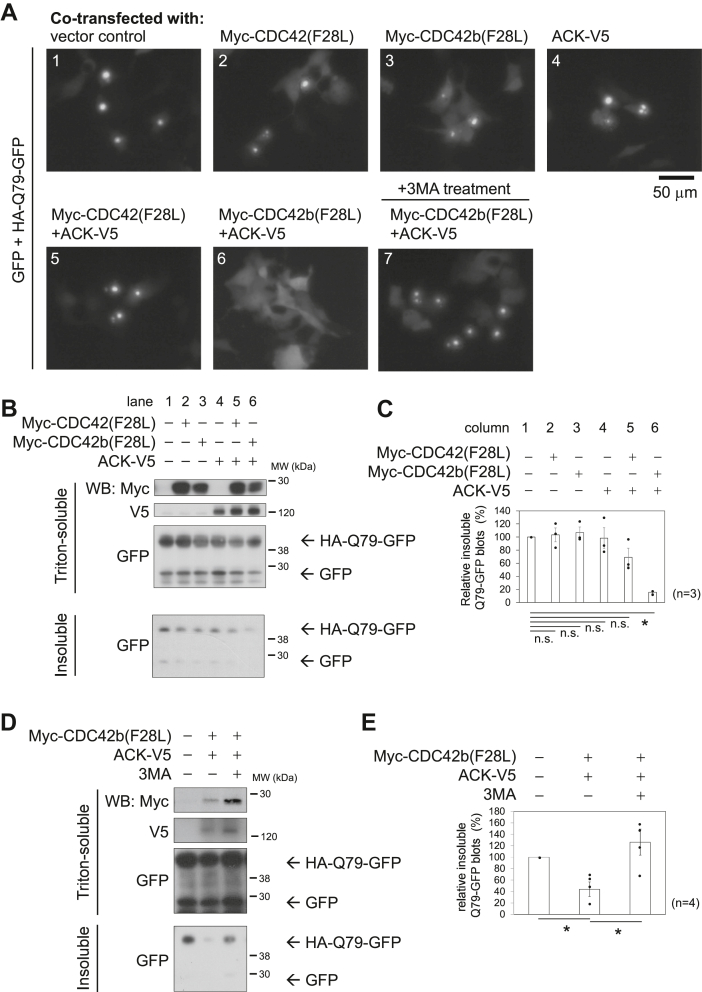
**Cdc42b and ACK promote autophagy-dependent degradation of aggregated polyglutamine-tagged proteins.***A*, epifluorescence live images showing the aggregation of HA-tagged polyglutamine (79 glutamine chain)-containing GFP (HA-Q79-GFP). GFP and HA-Q79-GFP were expressed in COS7 cells in the presence and absence of the constitutively active mutants of Myc-tagged Cdc42 splice variants and ACK-V5. GFP and the soluble fraction of HA-Q79-GFP show ubiquitous distribution. Aggregated HA-Q79-GFP exhibits intense fluorescent signals, forming punctate structures. After transfection, cells were recovered with 10% FBS-containing DMEM for 5 h and then cultured for another 15 h in the presence or absence of 3MA. *Black bar* under the images indicates a scale (50 μm). *B*, immunoblotting images showing the Triton-soluble/Triton-insoluble fractions of GFP and HA-Q79-GFP. Vinculin served as a loading control. COS7 cells were transfected with DNA constructs expressing GFP and HA-Q79-GFP, in the presence or absence of the constitutively active mutants of Myc-tagged Cdc42 isoforms and ACK-V5, and cultured in 10% FBS-containing DMEM for 20 h. Cell lysates were fractionated into Triton-soluble/Triton-insoluble populations and subjected to further analysis. *Black bars* and numbers beside the panels indicate the positions and molecular sizes (kDa) of molecular markers. *C*, histograms showing the relative amounts of Triton-insoluble HA-Q79-GFP. The levels of Triton-insoluble HA-Q79-GFP were quantified by densitometry and normalized to the amounts of Triton-soluble GFP. The normalized levels were presented relative to that of control vector-transfected cell lysates. Error bars indicate SEM (n = 3). Significance of differences is indicated by n.s. (not significant, *p* > 0.05) and ∗ (*p* < 0.05) using a *t* test. *D*, immunoblotting images showing the Triton-soluble/Triton-insoluble fractions of GFP and HA-Q79-GFP, with/without ACK-V5, Myc-tagged CDC42 proteins, and 3MA. *E*, histograms show the relative amounts of Triton-insoluble HA-Q79-GFP. Error bars indicate SEM (n = 4). Significance of differences is indicated by n.s. (not significant, *p* > 0.05) and ∗ (*p* < 0.05) using a Tukey’s test. 3MA, 3-methyladenine; FBS, fetal bovine serum.

To examine the effects of the CDC42 splice variants and ACK on autophagic activity, either Myc-CDC42(F28L), Myc-CDC42b(F28L), or ACK-V5 was expressed together with HA-Q79-GFP. GFP was also expressed as a control to monitor transfection efficiency and the selectivity of protein degradation. When solely expressing either Myc-CDC42(F28L), Myc-CDC42b(F28L), or ACK-V5, aggregated HA-Q79-GFP showed punctate fluorescent signals, as was the case in vector control cells, suggesting that the individual expression of these proteins did not significantly alter autophagic activities in COS7 cells (Fig. 2*A*, panels 1–4). The combination of Myc-CDC42b(F28L) and ACK expression diminished the punctate fluorescent signals caused by HA-Q79-GFP aggregates while not affecting the background GFP signals (Fig. 2*A*, panel 6), whereas the coexpression of Myc-CDC42(F28L) and ACK-V5 did not affect the fluorescent puncta due to the HA-Q79-GFP aggregates (panel 5). The clearance of punctate aggregates caused by coexpressing Myc-CDC42b(F28L) and ACK-V5 was attenuated upon treatment with 3MA (panel 7), suggesting that Myc-CDC42b(F28L) and ACK-V5 promote the degradation of HA-Q79-GFP aggregates, as mediated through autophagic activities.

When GFP and HA-Q79-GFP were coexpressed in COS7 cells, most cytosolic proteins, including GFP and the soluble population of HA-Q79-GFP, were solubilized in 1% Triton-containing lysis buffer, whereas aggregated HA-Q79-GFP was solubilized only when boiled in 0.5% SDS-containing radioimmunoprecipitation assay (RIPA) buffer (Fig. 2*B*, lane 1). In 20 h after transfection, approximately 4% to 5% of HA-Q79-GFP was fractioned as Triton-insoluble aggregates (Fig. S2). The individual expression of either Myc-CDC42(F28L), Myc-CDC42b(F28L), or ACK-V5 was not sufficient to efficiently clear Triton-insoluble HA-Q79-GFP aggregates (Fig. 2*B*, lanes 2–4, and Fig. 2*C*, columns 2–4), while the combination of Myc-CDC42b(F28L) and ACK significantly reduced the amount of insoluble aggregates (Fig. 2*B*, lane 6, and Fig. 2*C*, column 6). This reduction did not affect the expression levels of GFP nor the soluble portion of HA-Q79-GFP, indicating the reduction in protein aggregates is processed in a highly selective process. Treatment with 3MA attenuated the effects on the clearance of HA-Q79-GFP aggregates caused by coexpressing Myc-CDC42b(F28L) and ACK-V5 (Fig. 2, *D* and *E*).

### CDC42b and ACK promote autophagy by promoting EGFR degradation

**Figure 3:**
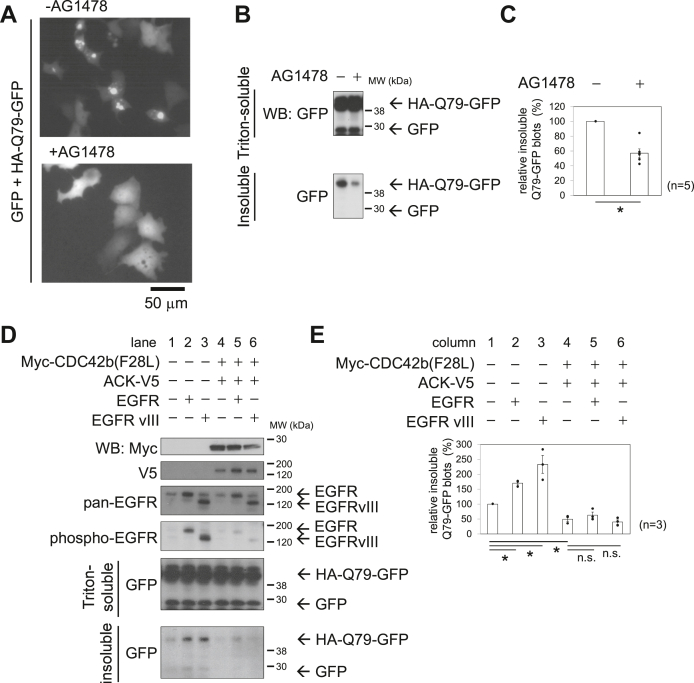
**CDC42b and ACK promote autophagy in an EGFR dependent manner.***A*, epifluorescence live images showing the aggregation of HA-Q79-GFP. COS7 cells were transfected with DNA constructs expressing GFP and HA-Q79-GFP. After transfection, cells were recovered with 10% FBS-containing DMEM for 5 h and then cultured for another 15 h, in the presence or absence of AG1478. *Black bar* under the images indicates a scale (50 μm). *B*, immunoblotting images showing the Triton-soluble/Triton-insoluble fractions of GFP and HA-Q79-GFP. *Black bars* and numbers beside the panels indicate the positions and molecular sizes (kDa) of molecular markers. *C*, histograms showing the relative amounts of Triton-insoluble HA-Q79-GFP. The levels of Triton-insoluble HA-Q79-GFP were quantified by densitometry and normalized to the amounts of Triton-soluble GFP. The normalized levels were presented relative to that of control vector-transfected cell lysates. *D*, immunoblotting images showing the expression and phosphorylation levels of the indicated proteins (*top four panes*) and the Triton-soluble/Triton-insoluble fractions of GFP and HA-Q79-GFP (*bottom two panels*). *E*, histograms showing the relative amounts of Triton-insoluble HA-Q79-GFP for the indicated conditions. Error bars indicate SEM (n = 5 in *C*, and n = 3 in *E*). Significance of differences is indicated by n.s. (not significant, *p* > 0.05) and ∗ (*p* < 0.05) using a *t* test (*C*) and a Tukey’s test (*E*). EGFR, EGF receptor; FBS, fetal bovine serum.

Since mTOR complex 1 (mTORC1) functions as a negative regulator of autophagy (39), this raised the possibility that CDC42b and ACK promoted autophagy by downregulating mTOR expression in the experiments described previously. However, EGF-dependent signaling pathways also negatively regulate autophagy by activating the class I PI-3 kinase, which is a major upstream activator of mTORC1, and indeed, EGFR inhibition leads to an enhancement of autophagy in certain types of cancer cells (41, 42). Therefore, we examined whether CDC42b and ACK regulate autophagy by downregulating EGFR as well as mTOR. To assess this possibility, we first examined how EGFR activation status affects autophagic activities. Upon treatment with an EGFR inhibitor, AG1478, the punctate fluorescent signals, characteristic of aggregated HA-Q79-GFP disappeared in COS7 cells (Fig. 3, *A*–*C*). Overexpression of WT EGFR or ectopic expression of an oncogenic EGFR mutant (EGFRvIII) increased the accumulation of Triton-insoluble HA-Q79-GFP aggregates in COS7 cells (Fig. 3*D*, lanes 1–3, and Fig. 3*E*, columns 1–3), most likely by attenuating the basal level of autophagic activity.

We next examined whether CDC42b and ACK enhanced the clearance of aggregation-prone proteins, as mediated through EGFR downregulation. Although coexpressing Myc-CDC42b(F28L) and ACK-V5 did not alter the overall protein levels of ectopically expressed EGFR and EGFRvIII, it caused a striking reduction in their phosphorylation levels (Fig. 3*D*, fourth panel from the top), suggesting that CDC42b and ACK preferentially downregulate the phosphorylated forms of these proteins. The coexpression of CDC42b and ACK was then sufficient to counteract the abilities of ectopically expressed EGFR or EGFRvIII to attenuate the clearance of aggregated HA-Q79-GFP (Fig. 3*D*, lanes 4–6, and Fig. 3*E*, columns 4–6).

### Suppressing EGF-dependent signaling activities is sufficient for promoting CDC42b- and ACK-dependent neuronal differentiation

**Figure 4:**
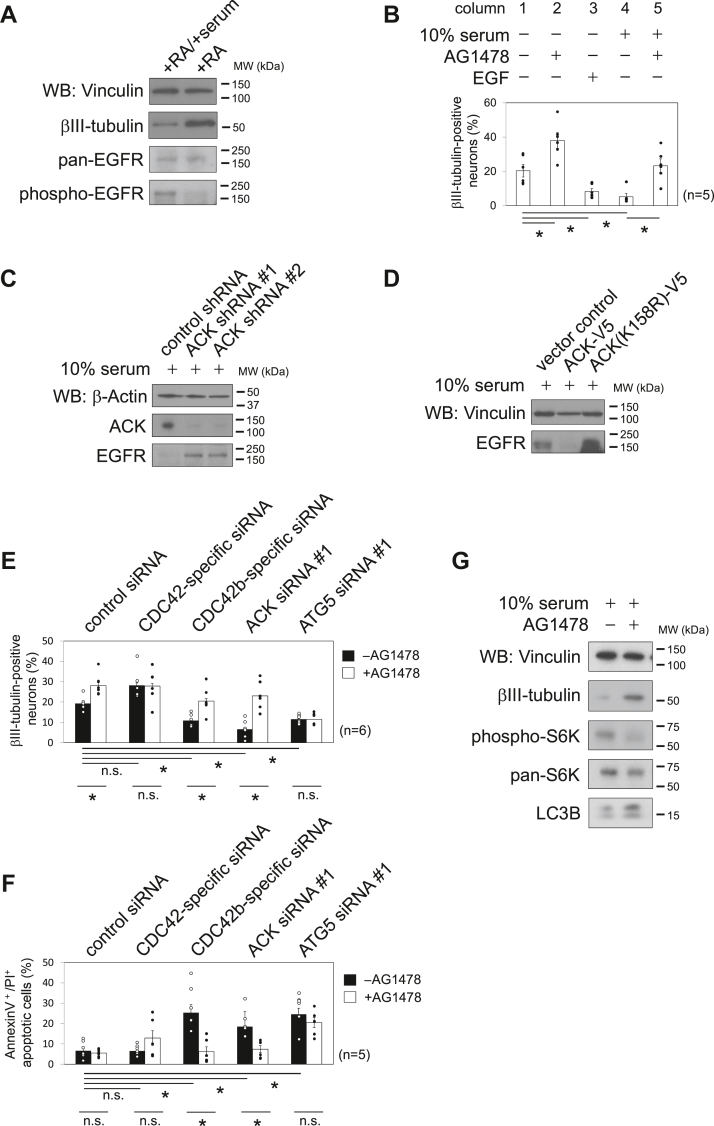
**Suppressing EGFR activity augments neural differentiation and autophagy.***A*, immunoblotting images showing the expression and phosphorylation levels of the indicated proteins. P19 cells were subjected to RA-induced neural protocol until day 6 and then cultured in 10% serum-containing αMEM (+RA/+serum) or B27-containing neurobasal media (+RA) until day 9. *B*, histograms showing the percentages of βIII-tubulin–positive neurons. P19 cells were subjected to RA-induced neural (−serum) or the neural progenitor cell (+10% serum) differentiation protocol in the presence or absence of AG1478 and EGF. Cells were fixed at day 9, and the numbers of βIII-tubulin–positive neurons were counted. Immunoblotting images showing the expression levels of the indicated proteins in ACK targeting shRNA-expressing (*C*) or ACK-V5-expressing cells (*D*). P19 cells were subjected to RA-induced neural progenitor cell differentiation. Histograms showing the percentages of βIII-tubulin–positive neurons (*E*) and apoptotic cells (*F*) upon treatment with siRNAs. Cells were subjected to transfection on day 5. Cells were fixed at day 9, and the numbers of βIII-tubulin–positive neurons or apoptotic cells were counted. Error bars indicate SEM (n = 5 in *B*, and n = 6 in *E* and *F*). Significance of differences is indicated by n.s. (not significant, *p* > 0.05) and ∗ (*p* < 0.05) using a Tukey’s test. *G*, immunoblotting images showing the expression and phosphorylation levels of the indicated proteins. P19 cells were subjected to the neural progenitor cell differentiation protocol in the presence or absence of AG1478. Cell lysates were collected at day 9. *Black bars* and numbers beside the panels indicate the positions and molecular sizes (kDa) of molecular markers. Vinculin (*A*, *D*, and *G*) or β-actin (*C*) served as loading controls. EGFR, EGF receptor; RA, retinoic acid.

Since EGF-dependent signaling pathways are involved in cell identity and proliferation in neural stem/progenitor cells (43), we considered whether CDC42b and ACK control neurogenesis by regulating EGFR homeostasis. Before addressing this possibility, we first examined the involvement of EGF-dependent signaling in the ability of embryonal carcinoma P19 cells to undergo neural differentiation. Upon treatment with RA, P19 cells first differentiated into Nestin-positive neural progenitor cells and then undergo terminal differentiation into βIII-tubulin–positive postmitotic neurons only when introduced to serum-free neurobasal media. Upon continuous treatment with 10% serum, P19-derived neural progenitor cells showed a reduced expression of βIII tubulin compared to cells treated with RA alone (Fig. 4*A*) but remained as Nestin-positive neural progenitors. Although EGFRs were equally expressed in serum-stimulated and serum-starved P19 cells on neural culture day 9, their phosphorylation level was significantly higher in serum-stimulated P19 cells than in serum-starved cells. In serum-starved P19 cells, treatment with AG1478 caused a significant increase in the number of βIII-tubulin–positive neurons (Fig. 4*B*, columns 1 and 2), whereas supplementation with EGF markedly downregulated βIII-tubulin expression (column 3). In the presence of serum, control cells did not undergo neuronal differentiation, whereas upon treatment with AG1478, a significant number of cells differentiated into βIII-tubulin–positive neurons (columns 4 and 5). These results demonstrate that suppressing EGF-dependent signaling pathways promotes neuronal differentiation from neural progenitors in P19 cells.

We next set out to examine whether ACK was involved in EGFR homeostasis during neural differentiation in P19 cells, as observed in non-neural epithelial cells. We first checked EGFR expression in ACK-targeting shRNA-expressing P19 cells. Expression of ACK shRNAs significantly increased the expression levels of EGFRs in P19-derived neural progenitors (Fig. 4*C*). We next examined EGFR expression in ACK-overexpressing P19 cells. In a stable cell line expressing ACK-V5, the EGFR expression was significantly decreased under neural progenitor culture conditions with 10% serum (Fig. 4*D*). In contrast, overexpression of a kinase-defective mutant of ACK, ACK(K158R)-V5, increased EGFR expression levels (Fig. 4*D*).

Since we previously found that knocking down CDC42b or ACK inhibits neuronal differentiation and causes apoptosis in P19 cells (12), we examined whether EGFR downregulation was sufficient for restoring the defects caused by knocking down CDC42b or ACK in P19-derived neurons. Knocking down CDC42b or ACK decreased the number of βIII-tubulin–positive neurons, whereas knocking down CDC42 slightly increased their numbers (Fig. 4*E*, *black* columns). Upon treatment with AG1478, the number of βIII-tubulin–positive neurons increased in CDC42b- or ACK-knocked down cells (Fig. 4*E*, *white* columns) and showed normal neurite extension (Fig. S3, panels 1–4 and 6–9). Knocking down CDC42b or ACK resulted in an increased number of apoptotic cells, due to an inhibition of autophagy, whereas knocking down CDC42 showed no significant effects (Fig. 4*F*, *black* columns). Treatment with AG1478, by blocking EGFR kinase activity, decreased the number of apoptotic cells caused by knocking down CDC42b or ACK (Fig. 4*F*, *white* columns).

### Autophagic activities are essential for neuronal differentiation

The results described in the preceding section raised a question as to which signaling pathway downstream of the EGFR was responsible for the AG1478-induced augmentation in neuronal differentiation in P19 cells. Among the cell signals that we tested, we were particularly interested in the changes in S6 kinase (S6K) and LC3B that were caused by treatment with the EGFR tyrosine kinase inhibitor AG1478. We observed that treatment with AG1478 decreased the phosphorylation level of S6K, a downstream target of mTORC1 (Fig. 4*G*), indicating that EGFR inhibition might enhance autophagic activities during AG1478-induced neurogenesis. Indeed, treatment with AG1478 upregulated the expression level of LC3B (Fig. 4*G*), a protein linked to autophagic activity.

**Figure 5:**
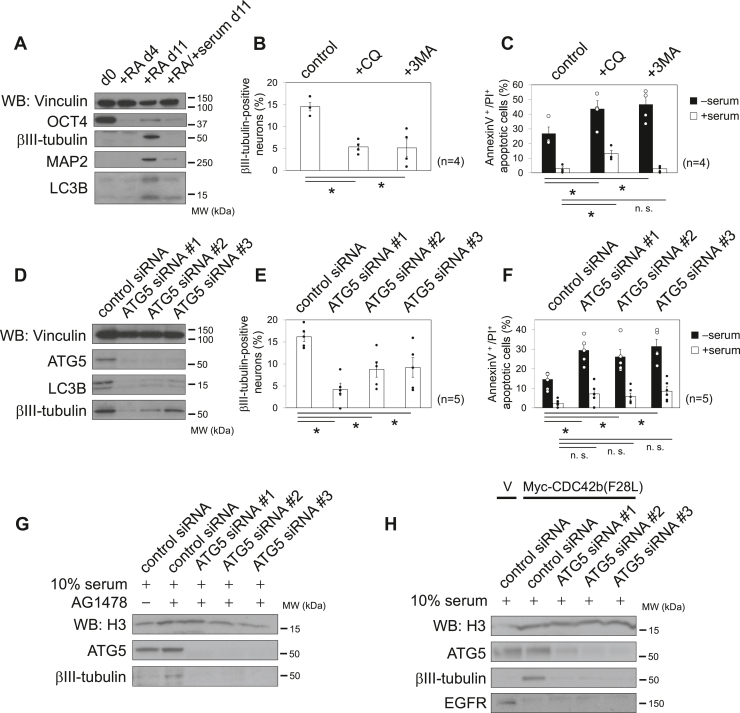
**Autophagic activities are essential for neuronal differentiation.***A*, immunoblotting images showing the expression levels of the indicated proteins. Cell lysates were collected at the indicated days before (d0) or after P19 cells were subject to RA-induced neural protocol. *B*, histograms showing the percentages of βIII-tubulin–positive neurons upon treatment with CQ and 3MA. P19 cells were subjected to the RA-induced neural differentiation protocol until day 6 and then treated with CQ or 3MA until day 9. Cells were fixed, and the numbers of βIII-tubulin–positive neurons were counted. *C*, histograms showing the percentage of apoptotic cells. Cells were fixed at day 9, and the numbers of apoptotic cells were counted. *D*, immunoblotting images showing the expression levels of the indicated proteins. P19 cells were subjected to the RA-induced neural differentiation protocol. Cells were transfected with siRNAs at day 5, and cell lysates were collected at day 9. Histograms showing the percentages of βIII-tubulin–positive neurons (*E*) and apoptotic cells (*F*), upon treatment with siRNAs. Error bars indicate SEM (n = 4 in *B* and *C*, and n = 5 in *E* and *F*). Significance of differences is indicated by n.s. (not significant, *p* > 0.05) and ∗ (*p* < 0.05), using a *t* test. *G* and *H*, immunoblotting images showing the expression levels of the indicated proteins. Parental P19 cells were subjected to the RA-induced neural progenitor differentiation protocol in the absence or presence of AG1478 (day 6–9) (*G*). P19 stable cell lines (V, vector control cells; Myc-CDC42b(F28L), Myc-CDC42b(F28L)-expressing cells) were subjected to RA-induced neural progenitor differentiation protocol (*H*). Cells were transfected with siRNAs at day 4, and cell lysates were collected at day 9. *Black* bars and numbers beside panels indicate the positions and molecular sizes (kDa) of molecular markers. Vinculin (*A* and *D*) or Histone 3 (H3) (*G* and *H*) served as a loading control. 3MA, 3-methyladenine; CQ, chloroquine; RA, retinoic acid.

Autophagy has been suggested to be essential for embryonic neurogenesis and neural homeostasis during adulthood (39, 44). However, it remains unknown how autophagic activities affect the transition from neural progenitor cells into postmitotic neurons. Before further exploring the importance of autophagy in AG1478-induced neurogenesis, we first examined its role in neural differentiation in P19 cells. The expression levels of LC3B were low and unchanged in RA-stimulated P19 cells on neural culture day 4 (Fig. 5*A*, indicated as +RA d4), compared with OCT4-positive undifferentiated cells (d0). The LC3B expression level remained low in neural progenitor cells (+RA/+serum day 11), while it was significantly increased in βIII-positive neurons (+RA day 11), suggesting that autophagic activities increase during neuronal differentiation. To further examine the importance of autophagy in neural differentiation in P19 cells, we suppressed autophagic activities, using two chemical inhibitors, 3MA and a lysosomal inhibitor chloroquine (CQ). Treatment with 3MA or CQ inhibited the upregulation of βIII-tubulin expression (Fig. 5*B*) and increased the number of apoptotic cells under serum-starved conditions (Fig. 5*C*, *black* columns). Treatment with 3MA or CQ did not significantly affect the number of apoptotic cells in neural progenitor cells to the same extent as in neurons (Fig. 5*C*, *white* columns), suggesting a specific role for autophagic activities in neuronal differentiation.

We then performed RNAi experiments to suppress autophagy in P19 cells. Knocking down ATG5 inhibited the upregulation of LC3B and βIII-tubulin expression in RA-stimulated P19 cells under neuronal culture conditions (Fig. 5*D*). Upon treatment with ATG5 siRNAs, P19 cells showed a lesser ability to differentiate into βIII-tubulin–positive neurons (Fig. 5*E*). As we observed in the experiments using chemical inhibitors, the suppression of autophagic activities using ATG5-targeting siRNAs increased the number of apoptotic cells under neuronal culture conditions (*i*.*e*., in the absence of serum) but not in the presence of 10% serum (Fig. 5*F*). Collectively, these findings support the idea that autophagic activities are essential for neural progenitor cells to differentiate into postmitotic neurons.

We next examined whether autophagy was essential for the augmented neurogenesis induced by AG1478. Under serum-starved neuronal culture conditions, treatment with AG1478 did not increase the number of βIII-tubulin–positive neurons in ATG5 siRNA-treated cells, unlike control siRNA-treated cells (Fig. 4*E*), nor did it restore the defects to neurite extension or present the increase in apoptosis that occurred in ATG5 siRNA-treated cells (Fig. S3, panels 5 and 10, and Fig. 4*F*). In the presence of serum, knocking down ATG5 also inhibited AG1478-induced upregulation of βIII-tubulin (Fig. 5*G*), thus further supporting the idea that blocking EGFR tyrosine kinase activity promotes neuronal differentiation through an autophagy-dependent mechanism.

We previously showed that the ectopic expression of CDC42b(F28L) promotes neuronal differentiation even in neural progenitor culture conditions (12). Therefore, we next examined whether autophagy was essential for the augmented neurogenesis induced by the constitutively active CDC42b(F28L). Expression of Myc-CDC42b(F28L) upregulated the expression of βIII-tubulin and downregulated EGFR expression in the presence of serum, unlike vector control cells (Fig. 5*H*). Treatment with ATG5 siRNAs then suppressed the upregulation of βIII-tubulin, induced by the expression of Myc-CDC42b(F28L) (Fig. 5*H*), thereby demonstrating that Myc-CDC42b(F28L)-induced neurogenesis occurs through autophagy.

### CDC42 splice variants exhibit distinct plasma membrane localizations

**Figure 6:**
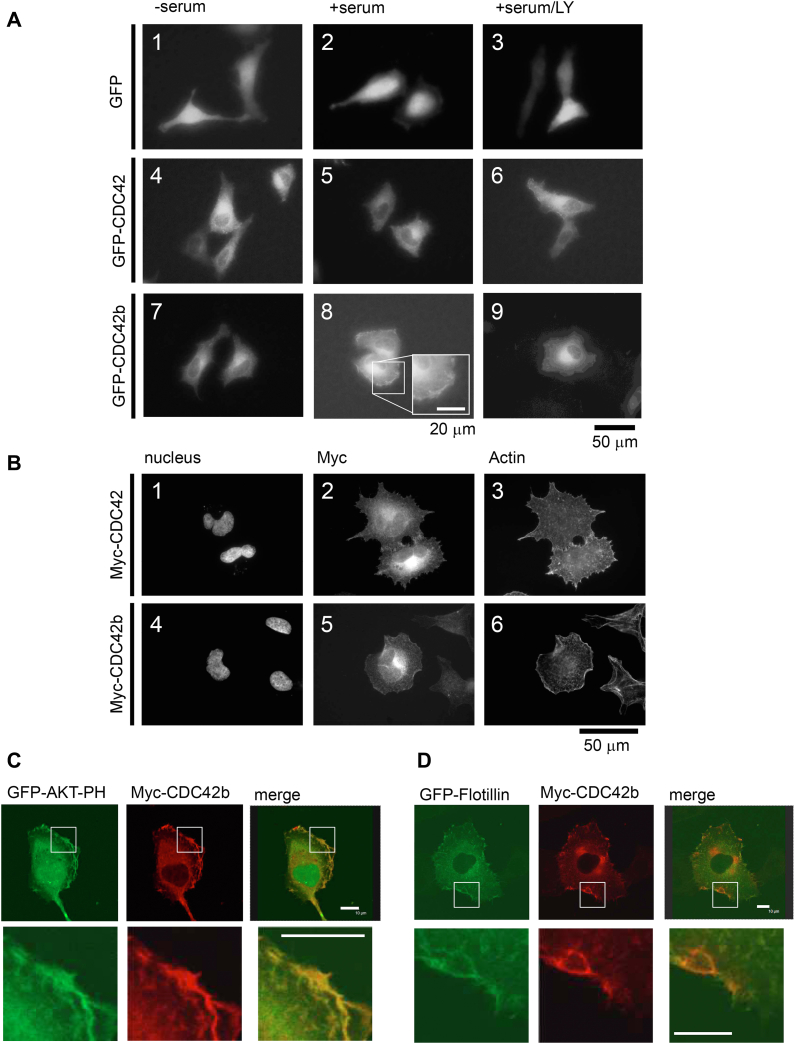
**CDC42 splice variants exhibit distinct plasma membrane localizations.***A*, epifluorescence live images showing subcellular localization of GFP or GFP-tagged CDC42 splice variants. HeLa cells were transfected with DNA constructs expressing GFP or GFP-tagged CDC42 splice variants. After transfection, cells were cultured in 10% FBS-containing DMEM for 18 h. On the next day, cells were serum starved for 5 h in the presence or absence of LY294002 (LY). After serum starvation, cells were stimulated with 10% FBS-containing DMEM for 15 min in the absence or presence of LY. *Black* bar under the images indicates a scale (50 μm). *White bar* in the inset indicates a scale (20 μm). *B*, epifluorescence images showing subcellular localizations of Myc-tagged CDC42 splice variants and actin structures in HeLa cells. Transfected HeLa cells were fixed and immunostained with anti-Myc antibody. Nuclei and actin structures were visualized using Hoechst and Texas Red X-phalloidin staining. *Black bar* under the images indicates a scale (50 μm). *C* and *D*, confocal immunofluorescence images showing the colocalizations of CDC42b with marker proteins in cell peripheral regions. HeLa cells were transfected with DNA constructs expressing Myc-tagged CDC42b, GFP-AKT-PH (PIP_3_ marker) (*C*), and GFP-Flotillin (cholesterol-enriched microdomain marker) (*D*). *White bars* in the merged images and insets indicate a scale (10 μm). FBS, fetal bovine serum.

Our previous and current studies clearly show that CDC42 splice variants are not functionally redundant, even with the slight differences in their HRVs (12). Since the HVRs of small GTPases are essential for determining their membrane localizations, we next examined the subcellular localizations of CDC42 splice variants. HeLa cells were transfected with DNA constructs expressing GFP or GFP-tagged CDC42 splice variants. After 18 h of recovery, cells were serum starved for 5 h and then stimulated with 10% fetal bovine serum (FBS)–containing Dulbecco's modified Eagle's medium (DMEM) for 15 min. In live-imaging fluorescent microscopy, GFP was distributed into both nuclei and cytosols (Fig. 6*A*, panels 1 and 2) and did not show any specific subcellular localization, while GFP-CDC42 showed perinuclear localization (panels 4 and 5), as previously shown (32). Upon stimulation with serum, GFP-CDC42b was localized in perinuclear regions and exhibited more significant cell peripheral localization than GFP-CDC42 (panels 7 and 8). Although epifluorescence microscopic images of fixed cells with higher magnification revealed the plasma membrane (PM) localization of Myc-tagged CDC42 that we did not observe in live-imaging microscopy, the membrane distribution of Myc-CDC42 was different from that of Myc-CDC42b. Myc-CDC42 showed thin spike or punctate distributions on the PM (Fig. 6*B*, panel 2), while Myc-CDC42b was accumulated in leading edges (panel 5). The costaining with Texas Red-phalloidin revealed that Myc-CDC42 induced filopodial actin structures (panel 3), while Myc-CDC42b induced lamellipodia (panel 6). GST-PBD (p21-binding domain) pull-down assays confirmed that equal amounts of CDC42 splice variants were the GTP-bound form in this condition (Fig. S4), suggesting that the activation status of CDC42 splice variants does not account for the differences of their PM localizations and inducing actin structures.

Polybasic sequences of small GTPases determine their interactions with phosphoinositides (34), and we previously showed that CDC42 binds to PIP_2_ in its diarginine motif (Arg186 and Arg187) (38). In contrast, Myc-CDC42b did not show the colocalization with GFP-PLCδ-PH, a PIP_2_ marker (data not shown). Instead, Myc-CDC42b was colocalized with GFP-AKT-PH, a marker for PIP_3_-in lamellipodia (Fig. 6*C*). The cell peripheral localization of GFP-CDC42b was inhibited with a chemical inhibitor of phosphoinositide 3-kinase (PI3-K), LY294002 (Fig. 6*A*, panel 9). The perinuclear localizations of GFP-CDC42 and GFP-CDC42b were not affected by the treatment with LY294002 (panels 6 and 9).

A specific stimulant that we found to induce the cell peripheral localization of CDC42b was fibronectin. On fibronectin-coated dishes, GFP-CDC42b but not GFP-CDC42 showed strong cell peripheral localization, which was inhibited with LY294002 treatment (Fig. S5, panels 6, 7, 11, and 12). Myc-CDC42b showed the colocalization with GFP-Flotillin, a marker for cholesterol-enriched microdomains (Fig. 6*D*). Cholesterol forms microdomains/rafts on the PM by clustering in an actin polymerization-dependent manner (45). The depletion of cholesterol from the PM, using methyl-β-cyclodextrin (MβCD), inhibited fibronectin-induced cell peripheral localization of GFP-CDC42b (Fig. S5, panel 13), and it was restored with cholesterol replenishment (panel 14). The cell peripheral localization of GFP-CDC42b was inhibited with actin depolymerization using a chemical inhibitor cytochalasin D (panel 15). GFP and GFP-CDC42 did not show any difference in their subcellular localizations upon these treatments (panels 1–10). These results suggest that CDC42b is transported into PIP_3_-enriched microdomains on the PM in an actin polymerization-dependent manner.

### The polybasic sequences are essential for determining the PM localization of CDC42b

**Figure 7:**
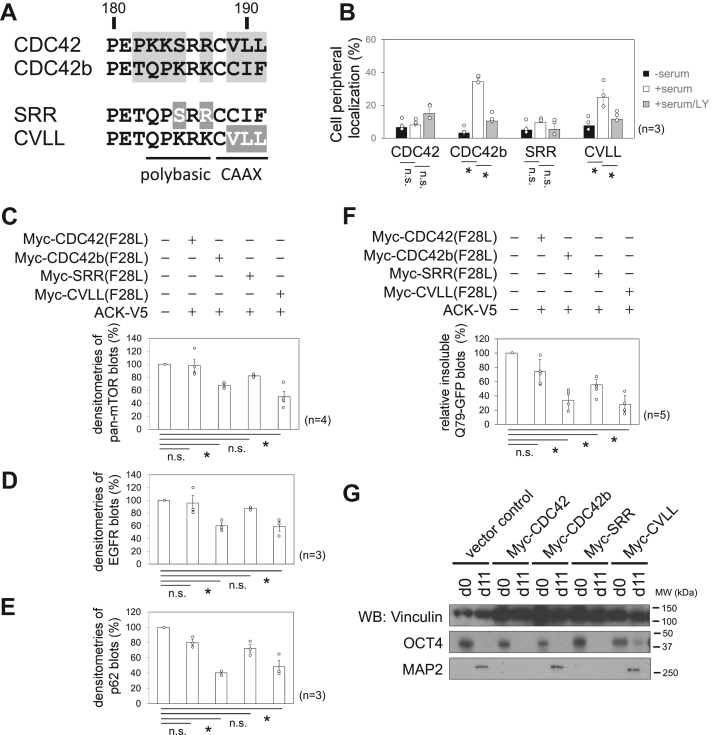
**The polybasic sequences are key to determining the functions of CDC42 splice variants.***A*, amino acid sequences of the hypervariable regions of CDC42 splicing variants and the CDC42b mutants used in this study. *Black* characters highlighted with *gray* background indicate different amino acid residues between CDC42 splice variants. *White* characters highlighted with *dark gray* background indicate point mutations of the CDC42b mutants. The positions of polybasic sequences and CAAX motifs are indicated with bars under the sequences. Bars and numbers above the sequences indicate the positions and numbers of amino acid residues. *B*, histograms showing the cell peripheral localization of GFP-tagged CDC42 proteins. HeLa cells were transfected with DNA constructs expressing GFP-tagged CDC42 proteins. After transfection, cells were cultured in 10% FBS-containing DMEM for 18 h. On the next day, cells were serum starved for 5 h in the presence or absence of LY294002 (LY). After serum starvation, cells were stimulated with 10% FBS-containing DMEM for 15 min in the absence or presence of LY. Cell numbers that showed cell peripheral localization of GFP-tagged CDC42 proteins were counted. Error bars indicate SEM (n = 3). Significance of differences is indicated by n.s. (not significant, *p* > 0.05) and ∗ (*p* < 0.05) using a *t* test. Histograms showing the expression levels of mTOR (*C*), EGFR (*D*), and p62 (*E*) in MDA-MB-231 cells in the presence or absence of Myc-tagged CDC42b mutants and ACK-V5. Error bars indicate SEM (n = 4 in C, n = 3 in *D* and *E*). *F*, histograms showing the relative amounts of Triton-insoluble HA-Q79-GFP. COS7 cells were transfected with DNA constructs expressing GFP and HA-Q79-GFP in the presence or absence of Myc-tagged CDC42b mutants and ACK-V5 and cultured in 10% FBS-containing DMEM for 20 h. Cell lysates were fractionated into Triton-soluble/Triton-insoluble populations and subjected to further analysis. The relative amounts of Triton-insoluble HA-Q79-GFP were compared by densitometry after they were normalized to Triton-soluble GFP. The normalized levels were presented relative to that of control vector-transfected cell lysates. Error bars indicate SEM (n = 5). Significance of differences is indicated by n.s. (not significant, *p* > 0.05) and ∗ (*p* < 0.05), using a *t* test. *G*, immunoblotting images showing the expression levels of differentiation marker proteins. Undifferentiated P19 stable cell lines (d0) were subjected to RA-induced neuronal differentiation protocol until day 11 (d11). Vinculin served as a loading control. *Black bars* and numbers beside the panels indicate the positions and molecular sizes (kDa) of molecular markers. EGFR, EGF receptor; FBS, fetal bovine serum; RA, retinoic acid.

CDC42 has been shown to be geranylgeranylated at the cysteine 188 residue of its CAAX motif (^188^CVLL^191^, Fig. 7*A*). Meanwhile, CDC42b contains two cysteine residues in its CAAX motif (^188^CCIF^191^), and previous studies show that both cysteine residues are subject to palmitoyl modification (35, 36, 37). In addition, Cys188 of CDC42b has been suggested to be subject to geranylgeranylation and farnesylation, as well (36, 46). Therefore, we next examined the effects of lipid modifications to the subcellular localizations of CDC42 splice variants. Geranylgeranyl transferase inhibitor-2133 (GGTI), farnesyl transferase inhibitor-277 (FTI), and 2-bromopalmitate (2BP) inhibit geranylgeranylation, farnesylation, and palmitoylation, respectively. Treatment with GGTI but not FTI nor 2BP inhibited the perinuclear localization of GFP-CDC42 (Fig. S6*A*, middle panels, and Fig. S6*B*). GFP-CDC42b exhibited perinuclear and cell peripheral localization in the same condition, and treatment with GGTI, FTI, and 2BP showed synergistic effects to inhibit the specific subcellular localization of GFP-CDC42b (Fig. S6*A*, bottom panels, Fig. S6, *C* and *D*). These results suggest that CDC42 is modified with geranylgeranyl moiety, while CDC42b can be modified with geranylgeranyl, farnesyl, and palmitoyl moieties, as suggested before (35, 36, 37, 46).

We next introduced a CDC42b mutant, CVLL, whose CAAX motif is swapped from the original sequence ^(188^CCIF^191^) into that of CDC42 (^188^CVLL^191^) (Fig. 7*A*). In the absence of the stimulation with fresh 10% FBS, GFP-CVLL exhibited perinuclear localization, as WTs CDC42b did (Fig. S7, panel 1). Upon treatment with GGTI but not FTI nor 2BP, GFP-CVLL lost the perinuclear localization (panels 2–4), suggesting that the CVLL mutant is subject only to geranylgeranylation, like CDC42. Upon stimulation with fresh 10% FBS, however, GFP-CVLL showed more significant cell peripheral localization than GFP-CDC42 in live-imaging microscopy and treatment with LY294002 inhibited it (Figs. 7*B* and S8, bottom panels).

To examine how the polybasic sequences contributed to the PM localizations of CDC42 splice variants, we introduced another CDC42b mutant, SRR, whose polybasic sequence was partially exchanged from the original one (^185^KRK^187^) into that of CDC42 (^185^SRR^187^) including the diarginine motif essential for the PIP_2_-binding of CDC42 (Fig. 7*A*) (38). Upon stimulation with serum, GFP-SRR exhibited perinuclear localization but did not show cell peripheral localization, regardless of the treatment with LY294002 (Figs. 7*B* and S8, top panels).

### The polybasic sequences are essential for determining the distinct biological functions of CDC42 splice variants

The results described previously show that the CAAX motif swapping mutant, CVLL, still maintains its ability to be localized on the PM in a PI3-K-dependent manner, as WT CDC42b does, while the polybasic swapping mutant, SRR, does not. Using these two swapping mutants, we next examined which of the CAAX motif or polybasic sequence is essential for the functional specification of CDC42 splice variants. In 231 cells, the coexpression of Myc-CVLL(F28L) with ACK significantly promoted the downregulation of mTOR, EGFR, and p62, while that of Myc-SRR(F28L) did not (Fig. 7, *C*–*E*). In COS7 cells, the coexpression of Myc-CVLL(F28L) with ACK cleared Triton-insoluble HA-Q79-GFP more efficiently than that of Myc-SRR(F28L) did (Fig. 7*F*). As previously shown (11, 12), the P19 embryonal carcinoma cell line that stably expresses Myc-CDC42 but not Myc-CDC42b failed to become neurons and upregulate the expression of MAP2 in RA-induced neuronal differentiation (Fig. 7*G*). Myc-SRR–expressing P19 cell line did not express MAP2, while Myc-CVLL–expressing one was capable to express MAP2, as vector control and Myc-CDC42b–expressing cells did (Fig. 7*G*). These results suggest that the differences of the polybasic sequences of CDC42 splice variants are essential for determining their abilities to regulate mTOR and EGFR degradation, autophagy, and neurogenesis.

## Discussion

**Figure 8:**
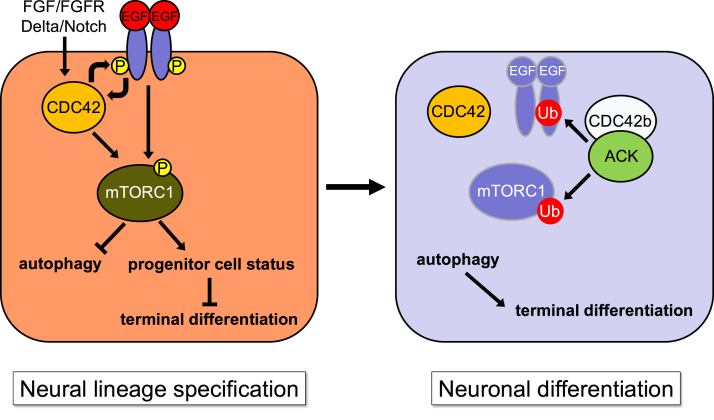
**Schematic drawing representing a model for the molecular mechanisms on how CDC42 splice variants and ACK regulate the transition of neural progenitor cells into postmitotic neurons (see details in the text).** P and Ub indicate protein phosphorylation and ubiquitination, respectively.

The Rho-family small GTPase CDC42 has been suggested to play a critical role in the development of the CNS, using conditional KO mouse models (6). Among the essential roles played by CDC42 are the establishment and maintenance of neuroepithelial and radial glial cells (8, 9, 10). In our previous studies, we showed that the CDC42 splice variants coordinate neural cell fate determination by regulating the expression of tissue-specific transcription factors, as mediated through the regulation of mTOR homeostasis (11, 12). Here, we now demonstrate how the EGFR serves as an important target of CDC42b and ACK in their roles to promote the transition of neural progenitor cells to terminally differentiate into neurons. (Fig. 8). Previously, we showed that the role of CDC42b in terminal neuronal differentiation likely involved additional signaling functions aside from negatively regulating mTOR activity. Indeed, the downregulation of mTORC1 activity, using rapamycin, was sufficient for reducing PAX6 expression (12) but not for completely restoring the defects to neurogenesis, caused by knocking down CDC42b or ACK. Our latest findings now point to the necessity of CDC42b working together with ACK to downregulate the EGFR, which is upstream of mTORC1.

The importance of autophagic activities has been well established in neural homeostasis during adulthood and is required for neuronal homeostasis because postmitotic neurons, which may survive for a lifetime, degrade dysfunctional organelles and protein aggregates, as mediated through autophagy (44). The biological functions of autophagy in embryonic neurogenesis are less clear, compared to the roles it plays during adulthood. One possible function of autophagy in embryonic neurogenesis is in maintaining cell survival during neuronal differentiation. Since neurons are cultured in serum-free media in conventional neuronal culture systems, autophagy might contribute to their adaption to the low nutrient environments by recycling metabolites, which is often observed in cancer cells under stressful conditions (41, 42). Another possibility is that autophagy is involved in cell differentiation directly, which is observed in other types of adult tissues. For example, in immune cells, autophagy promotes cell differentiation by clearing components used for the formation of original progenitor/precursor cells, such as transcription factors, cell signaling proteins, organelles, and metabolic enzymes, thereby enabling differentiating cells to efficiently change their cell status for functional specialization (47). Although it has not been well established in embryonic neural development, autophagy might contribute to a smooth transition during neural differentiation by promoting the replacement of cell components, as observed in the immune system.

An interesting question concerns the remarkable specificity exhibited by CDC42 *versus* CDC42b. By swapping different portions of the C-terminal hypervariable regions between these two GTPases, we found that the polybasic sequence, specifically ^185^KRK^187^ in CDC42b, is essential for specifying its unique PM localization and biological functions. We had previously reported that the diarginine motif (R186 and R187) of CDC42 is essential for binding with PIP_2_, inducing filopodia, and for inducing transformed phenotypes in fibroblasts by constitutively active CDC42 (38); while in this study, we show that the KRK motif is essential for the localization of CDC42b in PIP_3_-enriched microdomains on the PM, inducing lamellipodia. The differential signaling outputs of CDC42 and CDC42b then are likely due to their distinct capabilities for associating with or recruiting specific proteins to different membrane compartments. We recognize that other possible mechanisms exist for the underlying functional differences exhibited by the CDC42 splice variants, which we plan to examine in future studies. For example, electrostatic affinities of small GTPases with the PM can be modulated by the phosphorylation in the polybasic sequences. The S181 residue in K-RAS4B is phosphorylated by PKC, which modulates the electrostatic affinity with the PM and thereby regulate its biological functions (48). The S185 residue within the SRR sequence in CDC42 might also be subject to a phosphorylation to modulate its electrostatic affinity with the PM under certain conditions. Additionally, K183 and K184 in the HVR of CDC42, which is essential for the interaction with γCOP (49), have been shown to be subject to ubiquitination (50), which might affect the cellular behaviors of CDC42, as well.

Thus far, we have found that the differences in lipid modifications of CDC42 *versus* CDC42b do not account for their distinct functions, since the geranylgeranylated CDC42b mutant, CVLL, is still capable of promoting autophagy and neurogenesis. However, previous studies reported that palmitoylation is essential for the specific function of CDC42b to induce dendritic spine formation in neurons (35, 36, 37). We do not yet know the reasons for these contradictory results, although they may be due to differences in DHHC activities in the cells being examined in these studies or in the locations of CDC42b at different stages of neurogenesis. The palmitoylation of CDC42b in non-neuronal cells has been suggested to be relatively low, unless an exogenous DHHC protein is expressed (46). Additionally, we observed that RhoGDIα binds equally well to the two CDC42 splice variants in HeLa cells (unpublished data), although the palmitoylation of C189 attenuates the interaction of CDC42b with RhoGDIα (46). Therefore, differences in the extent of palmitoylation might then influence the cellular location and functional effects of CDC42b observed in different cellular contexts and in distinct stages of neurogenesis. For example, in an earlier phase of neurogenesis, geranylgeranylated CDC42b might promote autophagy and the transition of neural stem/progenitor cells into neurons, while at a later stage, a specific region of neurons where palmitoylation is more active, such as cholesterol-enriched dendritic protrusions, might enable CDC42b to be more efficiently palmitoylated by DHHC proteins, promoting the formation of dendritic spines.

It is still not clear how CDC42b is able to uniquely cooperate with ACK to regulate EGFR and mTOR homeostasis. Given that the ubiquitously expressed CDC42 is also capable of binding to this protein kinase, the locations where CDC42 splice variants interact with ACK are supposed to be key to understanding the mechanism underlying the functional differences of CDC42 splice variants. However, CDC42 splice variants did not show a clear colocalization with ACK in normal culture conditions that we tested (unpublished data). This is most likely because CDC42b and ACK are constantly subject to autophagic protein degradation in normal culture conditions, as indicated in Figures 1*F* and 2*D*, showing that an autophagic inhibitor, 3MA, increased the expression levels of Myc-CDC42b and ACK-V5. Further investigation is required to follow the life cycles and subcellular localizations of CDC42b and ACK. Additionally, the interaction of CDC42 with an effector other than ACK needs to be in consideration to explain the differences of CDC42 splicing variant to influence the ubiquitination of the EGFR. In this study, we observed that CDC42 reduced the ubiquitination levels of EGFRs induced by ACK, while CDC42b did not cause any obvious increase but nonetheless was required for EGFR degradation. Previously, we showed that CDC42 can reduce the ubiquitination of EGFRs in an ACK-independent manner, by forming a complex with the COOL-1/β-PIX and CBL, an E3 ligase for the EGFR, thereby preventing the EGFR from undergoing degradation (51). Therefore, through this mechanism, CDC42 might also counteract the ability of ACK to promote EGFR ubiquitination.

Although we used aggregate-prone polyglutamine-tagged proteins as a readout to examine autophagic activities in this study, we have not examined the pathological aspects of autophagic activities induced by CDC42b and ACK. The dysfunction of autophagic activities and the resultant accumulation of aggregate-prone proteins and damaged mitochondria are hallmarks of neurodegenerative diseases (44). Additionally, chronic activation of mTOR is observed in neurodegenerative diseases (52, 53). EGFR is also essential for the CNS homeostasis during adulthood, preventing neurodegeneration (43), and the inhibition of EGF-dependent cellular signaling activities has been suggested to be applicable as a therapeutic strategy to prevent or alleviate the accumulation of protein aggregations in neurodegeneration, as mediated through the upregulation of autophagy (54). Therefore, it will be interesting to see how the two forms of CDC42 affect the expression and/or activation levels of mTOR and the EGFR in adult brains and whether the deregulation of these CDC42 splice variants is involved in the pathogenesis responsible for neurodegenerative diseases.

## Experimental procedures

### Antibodies and reagents

Monoclonal anti-MAP2, anti-βIII tubulin (Tu20), and polyclonal anti-S6 kinase antibodies were purchased from Millipore. Monoclonal and polyclonal anti-p62 antibodies were from MBL International Corporation and Cell Signaling Technology, respectively. Monoclonal anti-OCT4 (C10), anti-ubiquitin (P4D1), anti-ACK (A11), and polyclonal anti-GFP (FL) antibodies were from Santa Cruz Biotechnology. Monoclonal anti-Vinculin antibody was from Sigma–Aldrich. Polyclonal or monoclonal antibodies that recognize LC3B, mTOR (total, 7C10; pSer 2448, D9C2), EGFR (total, 2232 and D38B1; pTyr 1068, 1H12), and S6 kinase (pThr389, 108D2) were obtained from Cell Signaling Technology. Monoclonal anti-Myc (9E10) and polyclonal anti-V5 antibodies were from Covance. GGTI-2133 (final concentration, 5 μM), FTI-277 (2 μM), LY294002 (4 μM), and AG1478 (2–4 μM) were from Calbiochem. Methyl-β-cyclodextrin (MβCD), cytochalasin D (2 μM), 2-bromopalmitic acid (2BP, 50 μM), CQ (20 μM), and 3MA (1 mM) were from Sigma–Aldrich. Cholesterol was from Nu-Chek Prep. EGF was from Invitrogen (final concentration, 10 ng/ml).

The mammalian expression DNA constructs were kindly provided from the following laboratories, mediated through Addgene: GFP-PLCδ-PH and GFP-C1-Akt-PH are from Dr Tobias Meyer lab, Stanford University (#21179 and #21218, respectively) (34); pHA-Q79-GFP was from Dr Junying Yuan, lab Harvard Medical School (#21159) (40). Constructs expressing carboxyl-terminal V5 tagged ACK (ACK-V5), Myc-tagged CDC42 and CDC42b proteins, the EGFR and EGFRvIII were generated as previously described (11, 12, 55). GFP-Flotillin was kindly provided from Dr David Holowka lab, Cornell University. Point mutants of CDC42b were generated using Quikchange II site-directed mutagenesis kit (Agilent Technologies). Constructs expressing GFP-tagged CDC42 and CDC42b proteins were generated using pEGFP-C1 vector by subcloning techniques.

### Cell culture, transfection, and immunoblotting

Cell culture and differentiation of P19 cells were performed as described (11, 12). P19-derived neural cells were treated with 4 μM of AG1478 for day 6 and then half of the concentration for days 7 to 9. HeLa and COS7 cells were cultured in 10% FBS-containing DMEM. MDA-MB-231 cells were cultured in 10% FBS-containing RPMI1640 medium. Transfections were performed using Lipofectamine-Plus reagent, Lipofectamine 2000, or Lipofectamine RNAiMax (Invitrogen), as suggested in the instructions from the manufacturer. Immunoblotting was performed as described previously (11, 12). In the protein ubiquitination experiment, cells were treated with CQ (20 μM) overnight after transfection to prevent protein degradation. Immunoprecipitation was performed using antibodies and Protein G agarose (Invitrogen), following the instructions from the manufacturer, in the presence of N-ethylmaleimide (10 mM), to prevent protein deubiquitination during the process. Immunoblot images were scanned, and densitometries were calculated using ImageJ (NIH). Establishing stable cell lines was performed as previously described (11, 12). To knock down ACK or ATG5 expression, Stealth RNAi predesigned siRNAs (Invitrogen) were used (ACK #1, MSS225066; ACK #2, MSS255067; ACK #3, MSS284822; ATG5 #1, MSS247019; ATG5 #2, MSS247020; ATG5 #3, MSS247021).

### Immunofluorescence microscopy

Epifluorescence microscopy was performed as described previously (11, 12). Briefly, transfected cells were placed on tissue culture plates for at least 12 h before capturing images. Cells were fixed with 3.7% formaldehyde, 2% paraformaldehyde, or ice-cold methanol and permeabilized with 0.1% Triton-X or 0.2% saponin (Sigma) containing PBS. After blocking with 2% bovine serum albumin, cells were stained with primary antibodies and subsequently, the secondary antibodies that recognize mouse or rabbit IgG, conjugated with fluorescence dyes (Molecular Probes). To visualize actin structures, cells were stained with Texas Red X-phalloidin, together with secondary antibodies. Epifluorescence images were captured with an Axioskop inverted microscope system (Carl Zeiss) equipped with a Sensicam qe charge-coupled device camera system (The COOKE Corp.) and processed with IPLab (SCANALYTICS Inc).

Epifluorescence live images of the subcellular localizations of GFP-tagged CDC42 proteins were captured with IX70 microscope system (Olympus) equipped with an AxioCam and processed with AxioVision (Carl Zeiss). Experimental conditions, including duration of the recovery after transfection, serum starvation, treatment with chemical inhibitors, and stimulation with serum or fibronectin, are indicated in the figure legends. Approximately, 50 or more cells were counted for each condition, and the percentage of perinuclear or cell peripheral localization of GFP proteins was determined.

Cholesterol depletion was performed by treating HeLa cells with 10 mM MβCD for 30 min before serum stimulation. For cholesterol replenishment, cells were incubated with 15 μg/ml cholesterol-0.15 mM MβCD for 1 h after the depletion. Epifluorescence live images were captured in each step.

Confocal fluorescence images were captured using Leica spectral confocal microscope system (Leica Microsystems) and Zeiss LSM510 Meta confocal microscope system (Carl Zeiss) in microscopy and imaging facility at Cornell University.

In neural differentiation assays, P19 cells were subjected to the RA-induced neural differentiation protocol and fixed on the indicated day. Cells were immunostained with anti-βIII–tubulin antibody. In neuronal differentiation assays, approximately 100 or more cells were counted for each condition, and the percentage of βIII-tubulin–positive cells was determined.

### Autophagic activity assays

Two-hundred and thirty-one cells were transfected with the indicated DNA constructs, using lipofectamine (Invitrogen), and after 18 to 21 h, cell lysates were collected and subjected to SDS-PAGE and Western blotting. The protein expression levels of p62 were monitored as a readout of autophagic activities. The expression levels of the *p62* mRNA were checked using semiquantitative PCR.

To monitor the autophagy-dependent degradation of polyglutamine-containing proteins, COS7 cells were transfected with pEGFP, pHA-Q79-GFP, and the indicated DNA constructs using lipofectamine and plus reagents (Invitrogen). After a 5 h recovery from the transfection with 10% FBS, cells were further cultured for 15 h, in the presence of 10% serum and the indicated chemicals. The transfected cells were subject to live-cell imaging microscopy, and epifluorescence images were captured with IX70 microscope system (Olympus) equipped with an AxioCam and processed with AxioVision (Carl Zeiss). Cells were then lysed with the lysis buffer (20 mM Hepes (pH7.5), 150 mM NaCl, 1% Triton X-100, 1 mM sodium orthovanadate, 20 mM NaF, 20 mM β-glycerophosphate, 10 μg/ml leupeptin and aprotinin), and cell lysates were subject to centrifugation. The supernatant served as a Triton-soluble fraction. The pellet was further washed with lysis buffer twice and then mixed with RIPA buffer (40 mM Hepes (pH7.5), 100 mM NaCl, 1% Triton X-100, 0.5% sodium deoxycholate, 0.5% SDS, 1 mM EDTA, 1 mM sodium orthovanadate, 25 mM β-glycerophosphate, 10 μg/ml leupeptin and aprotinin). The samples were vigorously vortexed and then boiled for 5 min. After further vortexing, the samples were subject to centrifugation. The RIPA-soluble supernatants served as a Triton-insoluble fraction. The samples were subject to SDS-PAGE and Western blotting. The blotting images were scanned, and densitometries of Triton-insoluble HA-Q79-GFP were normalized with those of Triton-soluble GFP.

### Apoptosis assay

Apoptotic cells were detected using the Annexin V: FITC apoptosis detection kit I (BD Bioscience), following the manufacture’s instruction. Annexin V-FITC^+^/propidium iodide^+^ apoptotic cells were counted using a fluorescence microscope system. Approximately, 100 or more cells were counted for each condition, and the percentage of apoptotic cells was determined.

### Statistical analysis

Statistical differences between two samples were calculated using a Student’s *t* test. Two-tailed *p* < 0.05 was considered to be significant. Statistical differences among more than two samples were calculated using a Tukey’s honestly significant difference test, after statistically significant differences among groups were calculated using a one-way ANOVA (*p* < 0.01). In a Tukey’s test, *p* < 0.05 was considered to be significant.

## Data availability

All data are contained within the article.

## Supporting information

This article contains supporting information.

## Conflict of interest

The authors declare that they have no conflicts of interest with the contents of this article.
