# Supplementary material for: The brain-specific splice variant of the CDC42 GTPase works together with the kinase ACK to downregulate the EGF receptor in promoting neurogenesis
Source: J Biol Chem. 2022 Oct 5;298(11):102564. doi: 10.1016/j.jbc.2022.102564 (PMC9663532; doi:10.1016/j.jbc.2022.102564)
Supplement: Supporting information [file mmc1.pdf]

The brain-specific splice variant of the CDC42 GTPase works together with the kinase ACK to down-regulate the EGF receptor in promoting neurogenesis

Makoto Endo and Richard A. Cerione

Supporting Information

Supporting Figure 1
Supporting Figure 2
Supporting Figure 3
Supporting Figure 4
Supporting Figure 5
Supporting Figure 6
Supporting Figure 7
Supporting Figure 8

Supporting Figure 1

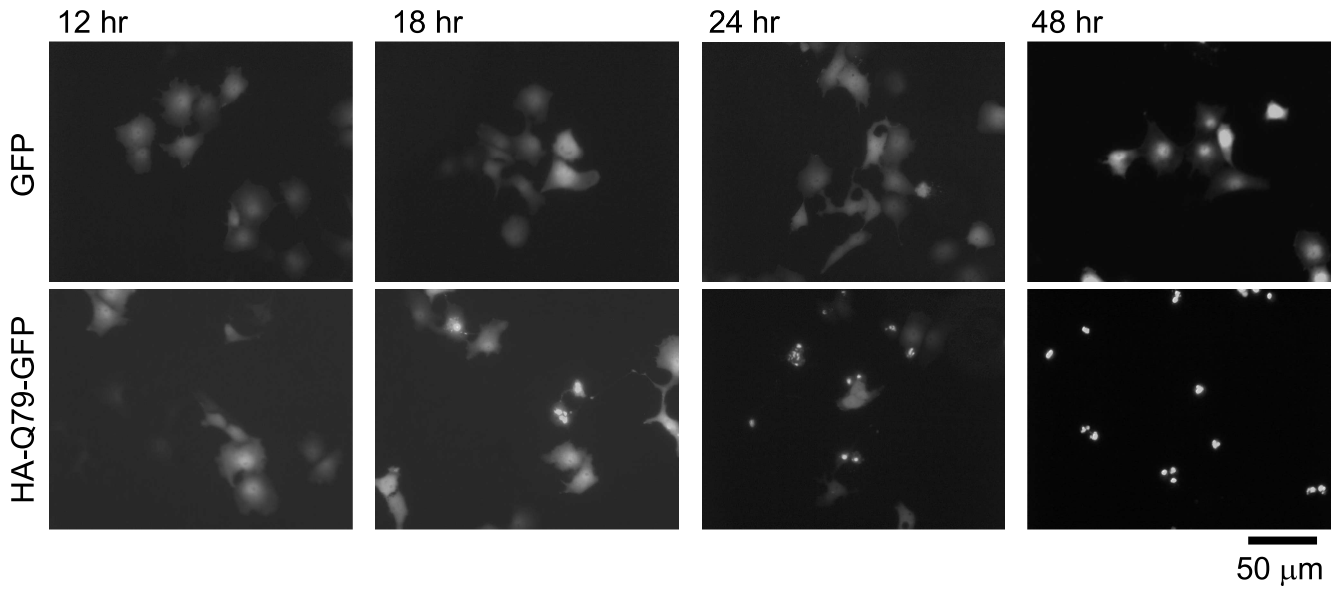

Supporting Figure 1

Epifluorescence live images showing GFP fluorescence and aggregations of HA-Q79-GFP. COS7 cells were transfected with DNA constructs expressing GFP (top panels) or HA-Q79-GFP (bottom panels) and then cultured for the indicated time. Black bar under the images indicates a scale (50 μm).

Supporting Figure 2

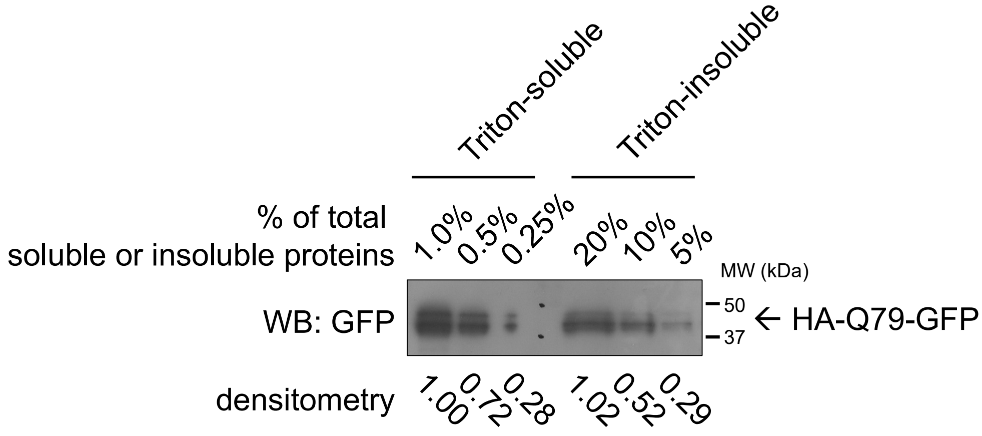

Supporting Figure 2

Immunoblotting image indicating the amounts of the Triton-soluble and insoluble HA-Q79-GFP proteins. COS7 cells were transfected with the DNA construct expressing HA-Q79-GFP. After transfection, cells were cultured in 10% FBS containing DMEM for 20 hours. The cell lysate was then collected and separated into Triton-soluble and -insoluble fractions. The indicated percentage of Triton-soluble or insoluble proteins was loaded into each well in an SDS-PAGE gel. The numbers below the image indicate densitometries of HA-Q79-GFP proteins**.** Black bars and numbers beside the panel indicate the positions and molecular sizes (kDa) of molecular markers. The quantitative ratio of Triton-soluble and insoluble HA-Q79-GFP proteins was estimated, based on the densitometries of HA-Q79-GFP proteins**.**

Supporting Figure 3

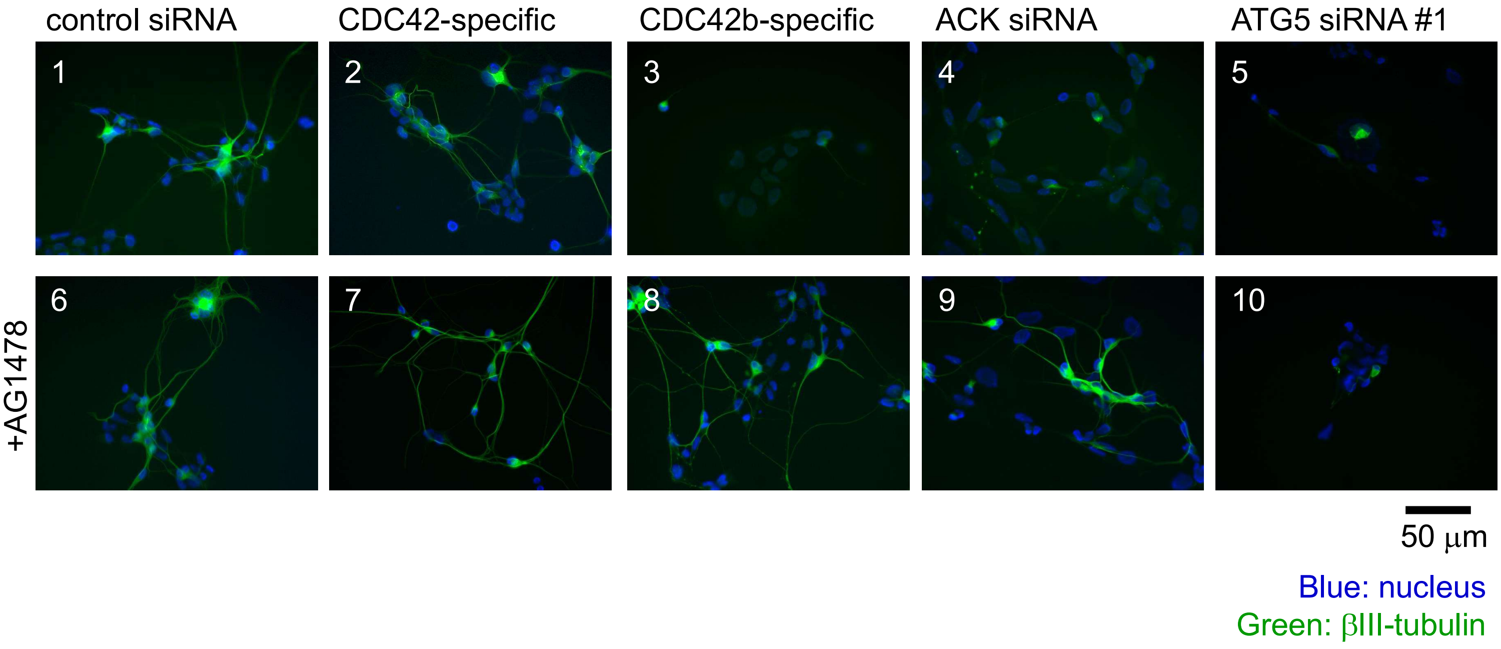

Supporting Figure 3

Epifluorescence images showing neurite extension of βIII tubulin-positive neurons, upon the indicated treatment. P19 cells were subjected to the RA-induced neuronal culture conditions. Cells were subjected to transfection on day 5, and then treated with chemical inhibitors from day 6 until day 9. Cells were fixed and then stained with anti-βIII tubulin antibody. Neurite extension status was observed based on βIII-tubulin staining. Black bar under the images indicates a scale (50 μm).

Supporting Figure 4

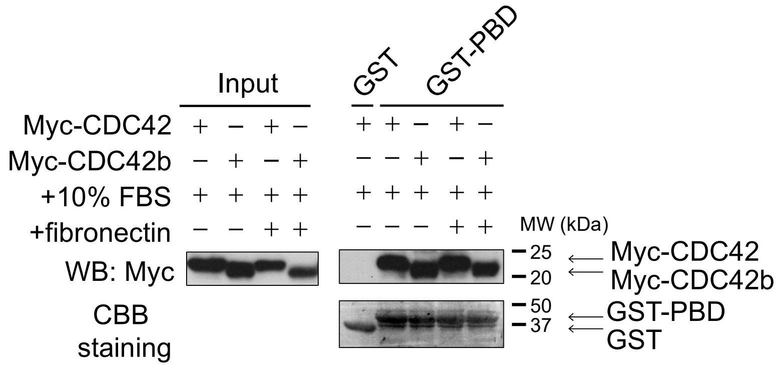

Supporting Figure 4

Immunoblotting images showing the activation status of the two CDC42 splice variants. HeLa cells were transfected with DNA constructs expressing Myc-tagged CDC42 proteins. After transfection, HeLa cells were cultured for 18 hours in non-coating or fibronectin-coating plates, in the presence of 10% FBS-containing DMEM. Cell lysates were subject to the PBD pull-down assays to detect the amount of the GTP-bound form of CDC42 proteins. The GST pull-down served as a negative control. The amounts of GST and GST-PBD proteins were detected with Coomassie Brilliant Blue (CBB) staining. Black bars and numbers beside the panels indicate the positions and molecular sizes (kDa) of molecular markers.

Supporting Figure 5

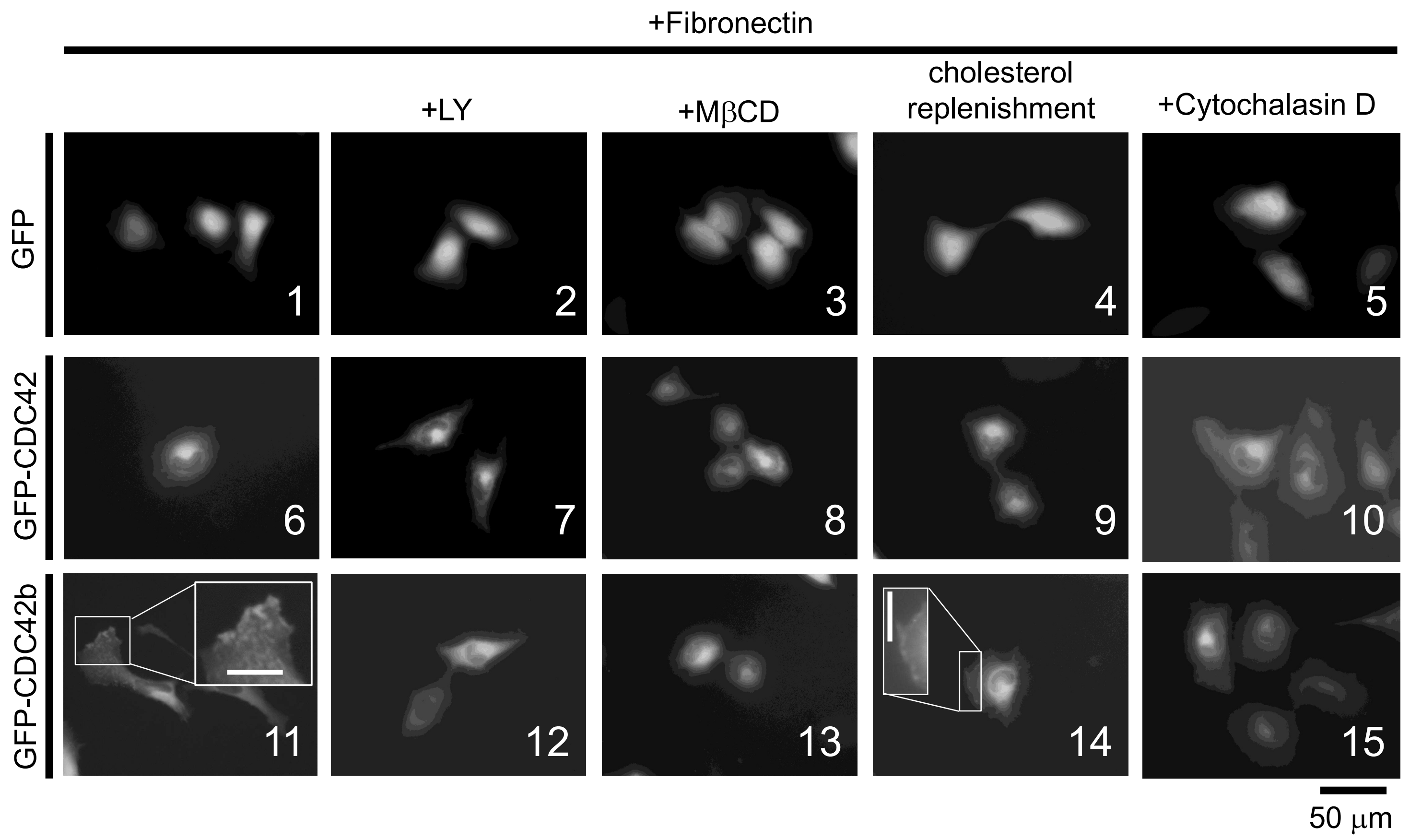

Supporting Figure 5

Epifluorescence live images showing the subcellular localization of GFP-tagged proteins in HeLa cells. HeLa cells were transfected with DNA constructs expressing GFP or GFP-tagged CDC42 splice variants. After transfection, HeLa cells were transferred to Fibronectin-coated dishes and cultured for 18 hours. Cells were then treated with LY294002 (LY) for 30 min or with cytochalasin D for 10 min before taking images. Cholesterol was depleted from cells by treating with methyl-β-cyclodextrin (MβCD) for 30 min, and then replenished by adding cholesterol for 1 hr. Cell peripheral localizations of GFP-tagged proteins were magnified in insets. Black bar under the images indicates a scale (50 μm). White bars inside the insets indicate a scale (20 μm).

Supporting Figure 6

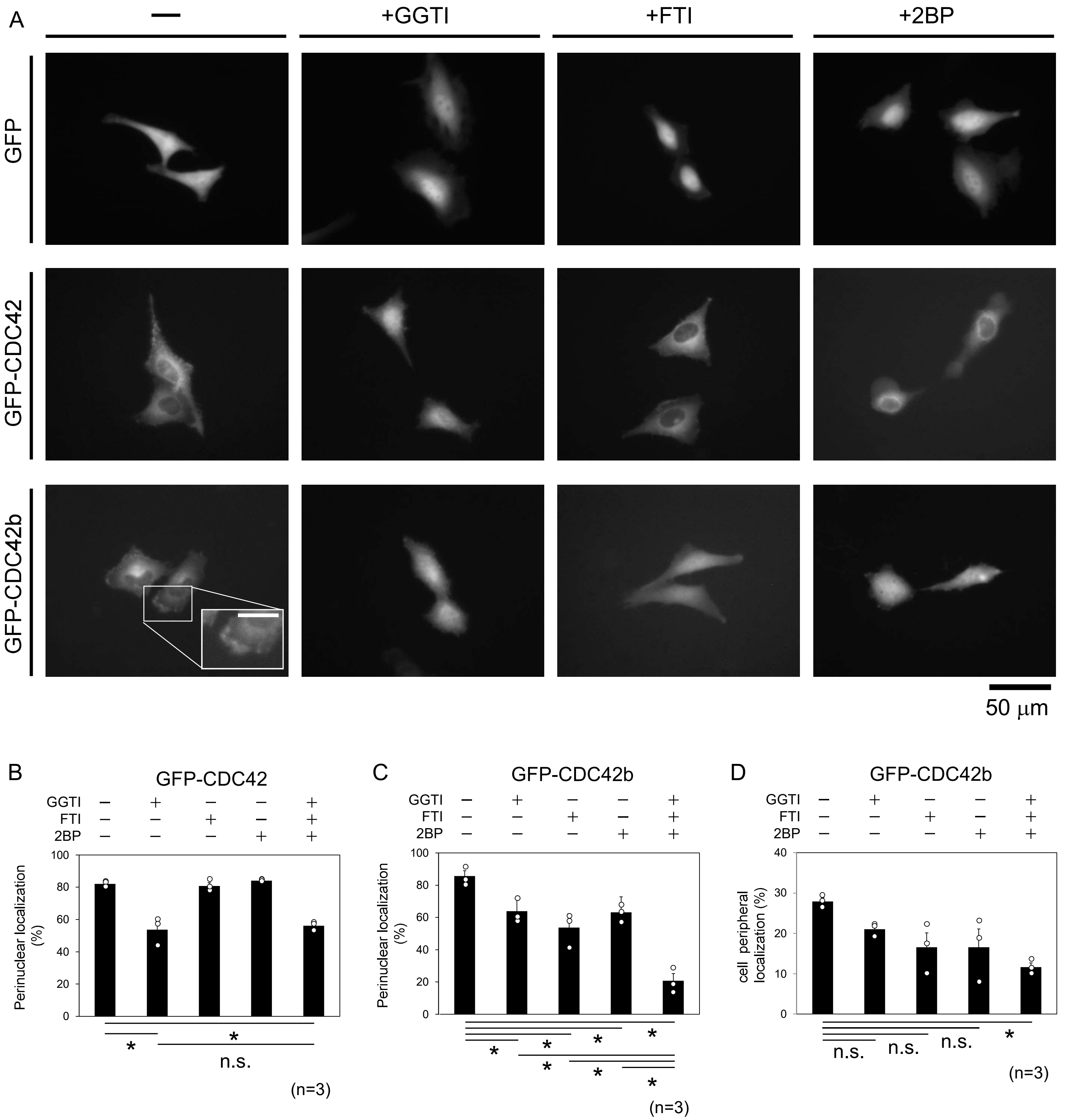

Supporting Figure 6

A, Epifluorescence live images showing the subcellular localization of GFP-tagged proteins in HeLa cells. HeLa cells were transfected with DNA constructs expressing GFP or GFP-tagged CDC42 splice variants. After transfection, cells were recovered with 10% FBS-containing DMEM for 5 hours and then cultured for another 18 hours, in the presence or absence of chemical inhibitors targeting lipid modification enzymes (GGTI-2231 for geranylgeranyltransferases, FTI-277 for farnesyltransferases, and 2BP for palmitoylation enzymes). On the next day, cells were stimulated with 10% FBS-containing DMEM for 15 min, in the presence or absence of inhibitors. Cell peripheral localizations of GFP-tagged proteins were magnified in insets. Black bar under the images indicates a scale (50 μm). White bar inside the inset indicates a scale (20 μm). Histograms showing inhibitory effects of chemical inhibitors to perinuclear (B and C) and cell peripheral localizations (D) of GFP-tagged CDC42 splice variants. Cell numbers that showed perinuclear or cell peripheral localization of GFP-tagged CDC42 splice variants were counted. Error bars indicate s.e.m. (n=3). Significance of differences is indicated by n.s. (not significant, $p>0.05$) and * ($p<0.05$) using a Tukey's (B and C) or *t*-test (D).

Supporting Figure 7

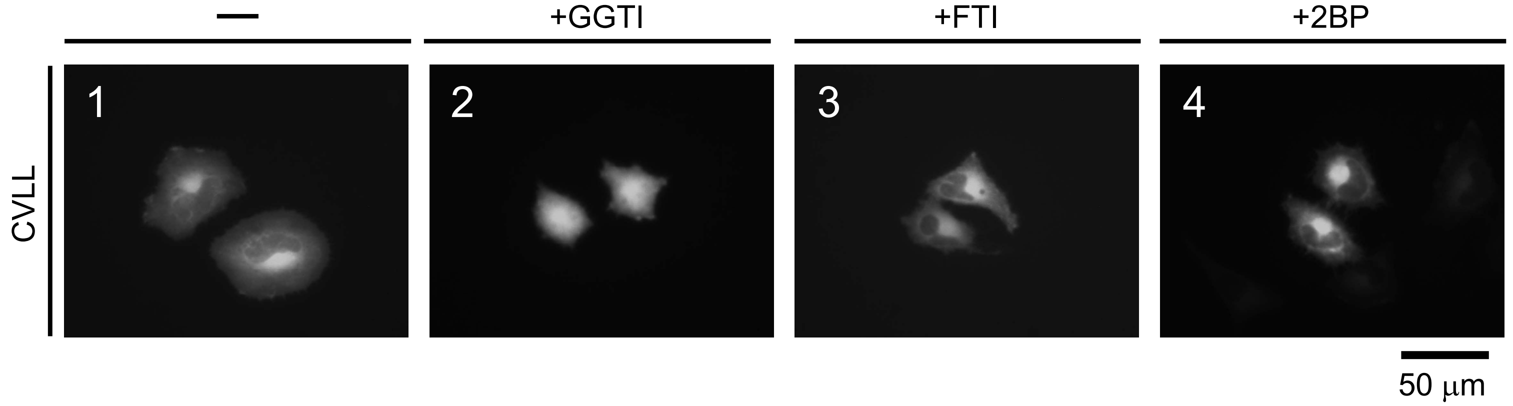

Supporting Figure 7

Epifluorescence live images showing the subcellular localization of the GFP-tagged CAAX motif mutant of CDC42b (CVLL) in HeLa cells. HeLa cells were transfected with the DNA construct expressing GFP-CVLL mutant. After transfection, cells were recovered with 10% FBS-containing DMEM for 5 hours and then cultured for another 18 hours, in the presence or absence of chemical inhibitors targeting lipid modification enzymes. Black bar under the images indicates a scale (50 μm).

Supporting Figure 8

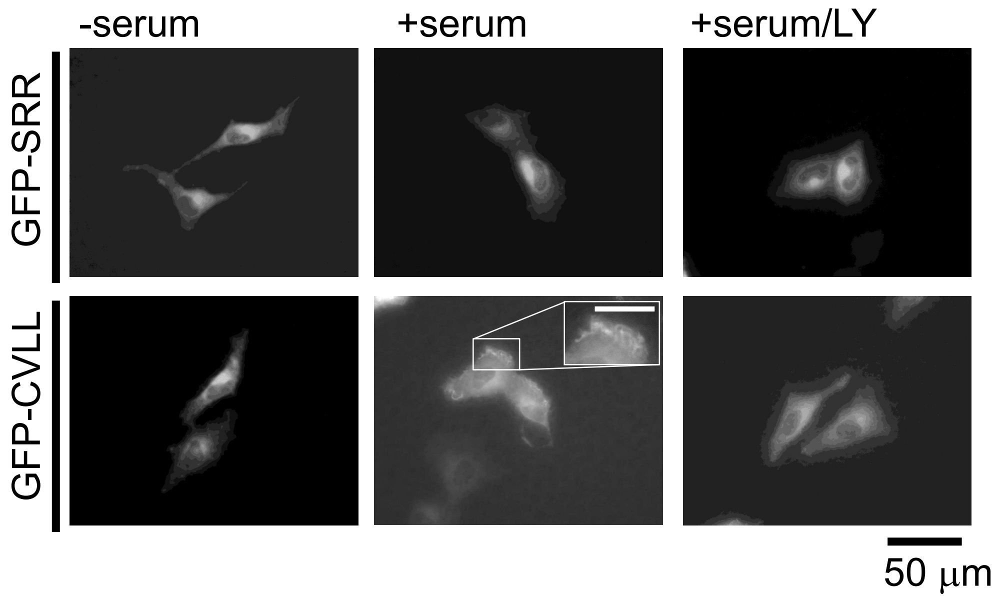

Supporting Figure 8

Epifluorescence live images showing subcellular localization of GFP-tagged CDC42b mutants. HeLa cells were transfected with DNA constructs expressing GFP-tagged CDC42b mutants. After transfection, cells were cultured in 10% FBS-containing DMEM for 18 hours. On the next day, cells were serum-starved for 5 hours, in the presence or absence of LY294002 (LY). After serum-starvation, cells were stimulated with 10% FBS-containing DMEM for 15 min, in the absence or presence of LY. Cell peripheral localization of GFP-CVLL was magnified in the inset. Black bar under the images indicates a scale (50 μm). White bar inside the inset indicates a scale (20 μm).
